# Gene Atlasing of Digestive and Reproductive Tissues in *Schistosoma mansoni*

**DOI:** 10.1371/journal.pntd.0001043

**Published:** 2011-04-26

**Authors:** Sujeevi S. K. Nawaratna, Donald P. McManus, Luke Moertel, Geoffrey N. Gobert, Malcolm K. Jones

**Affiliations:** 1 Queensland Institute of Medical Research, Herston, Australia; 2 School of Veterinary Sciences, The University of Queensland, Gatton, Australia; Uniformed Services University, United States of America

## Abstract

**Background:**

While considerable genomic and transcriptomic data are available for *Schistosoma mansoni*, many of its genes lack significant annotation. A transcriptomic study of individual tissues and organs of schistosomes could play an important role in functional annotation of the unknown genes, particularly by providing rapid localisation data and thus giving insight into the potential roles of these molecules in parasite development, reproduction and homeostasis, and in the complex host-parasite interaction.

**Methodology/Principal Findings:**

Quantification of gene expression in tissues of *S. mansoni* was achieved by a combination of laser microdissection microscopy (LMM) and oligonucleotide microarray analysis. We compared the gene expression profile of the adult female gastrodermis and male and female reproductive tissues with whole worm controls. The results revealed a total of 393 genes (contigs) that were up-regulated two-fold or more in the gastrodermis, 4,450 in the ovary, 384 in the vitelline tissues of female parasites, and 2,171 in the testes. We have also supplemented these data with the identification of highly expressed genes in different regions of manually dissected male and female *S. mansoni*. Though relatively crude, this dissection strategy provides low resolution localisation data for critical regions of the adult parasites that are not amenable to LMM isolation.

**Conclusions:**

This is the first detailed transcriptomic study of the reproductive tissues and gastrodermis of *S. mansoni*. The results obtained will help direct future research on the functional aspects of these tissues, expediting the characterisation of currently unannotated gene products of *S. mansoni* and the discovery of new drug and vaccine targets.

## Author Summary

There is currently only one drug available for treatment of *schistosomiasis mansoni* and no vaccine. The searches for possible new drug and vaccine candidates remain two major areas of current research in schistosomiasis. There are considerable amounts of data available on the genomics, transcriptomics and proteomics of *Schistosoma mansoni* from which useful candidates for future drug and vaccine development can be identified. Arranging these data into a biologically relevant context through the characterisation of gene expression profiles of the different tissues of this complex metazoan parasite, is an essential step in identifying molecules with potential therapeutic value. We have used laser microdissection microscopy and microarray analysis to show that many tissue-specific genes are up-regulated in the digestive and reproductive tissues of *S. mansoni*. This new knowledge provides an avenue to investigate the molecular components associated with fundamental aspects of schistosome biology.

## Introduction

The last decade has seen an exponential growth in the availability of genomic and proteomic data for *Schistosoma mansoni* and *S. japonicum* through genomic sequencing projects [1], [2] and numerous transcriptomic and proteomic studies [3], [4], [5], [6], [7], [8], [9]. However, the functions of many schistosome gene products remain unsolved. As argued by Jones et al [10], a major step towards elucidating function of these uncharacterised gene products lies in defining their sites of expression. Large-scale investigation of the expression repertoire of specific tissues is difficult to achieve because schistosome tissues generally cannot be isolated, due to their small tubiform bodies and the absence of a body cavity [10], [11], [12]. Laser micro-dissection microscopy (LMM), a tool that enables selection and sampling of small cell populations from histological sections, has been used to overcome the difficulty in separating the tissues for cell-enriched studies of these parasites [10], [13]. The approach has been used successfully by our team to study tissue specific transcriptomics of gastrodermal, ovary and vitelline tissues of adult females of *S. japonicum* [11].

In the current study, we describe the application of LMM, in conjunction with microarray gene expression analysis, to map the transcriptome of biologically important tissues of *S. mansoni*, augmenting our study on *S. japonicum* tissue specific transcriptomics [11]. The gastrodermis (absorptive gut lining) of females and the reproductive tissues of male (testes) and female (ovary, vitelline glands) worms were targeted with this gene atlasing strategy to determine the expression repertoire of these tissues.

## Materials and Methods

Research involving animals in this study was approved by the Animal Ethics Committee of the Queensland Institute of Medical Research. The study was conducted according to guidelines of the National Health and Medical Research Council of Australia, as published in the *Australian Code of Practice for the Care and Use of Animals for Scientific Purposes*, 7th edition, 2004 (www.nhmrc.gov.au).

### Sample preparation

The Puerto Rican strain of *Schistosoma mansoni* is maintained in ARC Swiss mice and *Biomphalaria glabrata* snails at QIMR from stocks originating from the National Institute of Allergy and Infectious Diseases Schistosomiasis Resource Centre, Biomedical Research institute (Rockville, Maryland, USA).

Worm pairs were perfused from mice six weeks post-infection. Males and females were separated and single-sex samples, consisting of 30–40 worms, were transferred immediately into Tissue-Tek Optimal Cutting Temperature compound (OCT) (ProSciTech, Thuringowa, Australia) and frozen in dry ice. Four OCT blocks, each containing 30–40 worms, were used for the microdissection of each sex of *S. mansoni*. Worm sections were prepared for microdissection as described [11]. Cryosections were cut onto PALM 0.17 mm polyethylene naphthalene membrane covered slides (Carl Zeiss Microimaging GmbH, Bernried, Germany).

### Laser microdissection of schistosome tissues

**Figure 1:**
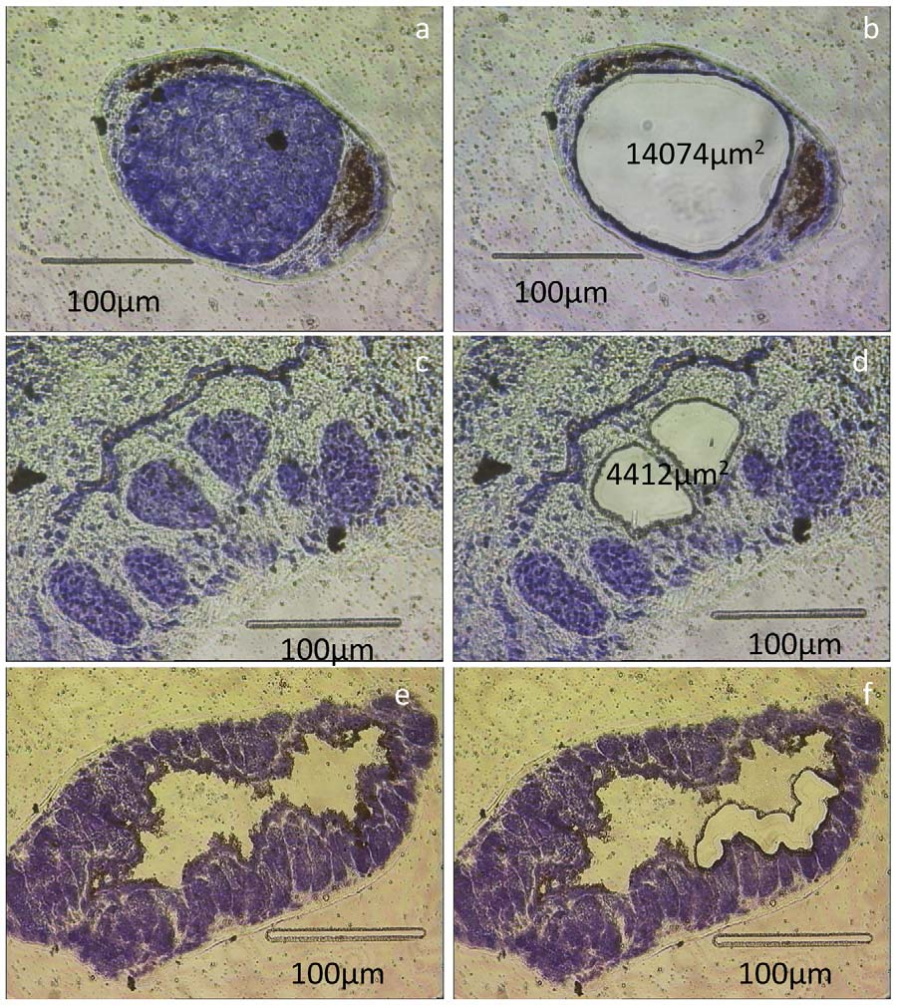
Laser Microdissection of adult *S. mansoni*. Sections of male and female *S. mansoni*, before and after microdissection, stained with 1% (w/v) toluidine blue. The left column shows the ovary (a), testes (c) and gastrodermis (e) before microdissection, while the right column shows the same sections of the ovary (b), testes (d) and gastrodermis (f) after microdissection.

Thawed cryosections of worms were stained with 1% (w/v) toluidine blue (CHROMA, Stuttgart, Germany), washed in distilled water and air dried in a sterile biohazard hood immediately prior to microdissection [11]. A PALM MicroBeam Laser Catapult Microscope (Carl Zeiss Microimaging) was used to microdissect the gut, ovary, vitelline glands of females and testes from males from the stained sections using a ×40 microdissection objective lens. The RoboLPC mode in PALMRobo 2.2.2 software (Carl Zeiss Microimaging) was used to cut and catapult the selected tissues from sections of adult schistosomes. Approximately 4×10^6^ µm^2^ of each tissue was catapulted into 200 µl opaque adhesive Teflon-coated caps (Carl Zeiss Microimaging) located in the laser path, a few millimetres above the specimen stage (Figure 1).

The caps containing the dissected material were stored at −80°C until used. Sex-matched controls were obtained by randomly microdissecting sections of female or male worms to obtain microdissected samples, representing the entire expression repertoire of the adult worms. For female controls, an area approximating those of the three target tissues was dissected randomly from sections of females. For males, it was necessary to dissect an area of 20×10^6^ µm^2^ in order to obtain sufficient sample material as control tissue.

### Macro-dissection

As a complement to the microdissection studies, a set of macro-dissected samples was also obtained from *S. mansoni* male and female worms using a dissecting microscope and sterile razor blade. Females were cut transversely into three parts: namely, the head region, extending from the anterior extremity of the schistosome to the ventral sucker; the middle region, extending from the posterior extremity of the ventral sucker to the posterior margin of the ovary; and the hind region, extending from the posterior extremity of the ovary to the posterior extremity of the parasite. The tissue types included in each of these regions are shown (Table S1). Because of its extremely small size, the head region of 100 females was dissected for RNA extraction, whereas the middle and hind regions were obtained from only 15 females. Males were dissected into two regions; namely, the head region, which was that extending from the anterior extremity of the schistosome to the ventral sucker, and the hind region, representing the remainder of the worm. Head region samples were dissected from 100 worms whereas the hind region was dissected from only 3 parasites. A total of 5 female and 3 male sex-matched controls were used. In each case, the worm number was determined by the maximum amount of tissue amenable for efficient RNA extraction using an RNAqueous-Micro kit (Ambion, Austin, TX, USA) (see below).

### Total RNA isolation and hybridisation

Total RNA was isolated from LMM samples and manually dissected samples using RNAqueous-Micro kits using the manufacturer's protocol for LMM samples and whole tissue samples, respectively. The quality of RNA was assessed using the Bioanalyser RNA Pico Lab Chip (Agilent Technologies, Santa Clara, CA, USA) as described [11].

A 44k oligonucleotide microarray platform (Agilent Technologies) designed specifically for *S. mansoni* was used for microarray analyses [14]. Access to the design was kindly provided by Dr Sergio Verjovski-Almeida (Universidade de São Paulo) [14]. The microarray platform has 39,342 probes representing 19,907 putative genes [14]. The array was designed using publicly available EST data for *S. mansoni* and *S. japonicum* to include orthologous *S. japonicum* genes not cloned and sequenced by the time of microarray construction [14]. The coverage and similarity of microarray used in this current study was compared to the microarray used in our previous reported study of *S. japonicum* [11], by performing a local BLASTn using BioEdit with default settings [15]. The BLASTn was performed with the ESTs used in the design of the original microarray used in the *S. japonicum* transcriptomic study, queried against the nucleotide database consisting of ESTs from the microarray used in the current study (Table S2).

Gene expression analysis was performed for all the samples and biological replicates. Two and three biological replicates were used for testes and vitelline tissue samples, respectively, and single samples were used for the rest of the tissues due to limited availability of material for analysis. Two technical replicates ( = two hybridisations) were performed for each sample.

Microarray hybridisation used 200 ng of RNA from each sample according to the manufacturer's instructions (One-colour Microarray-Based Gene Expression Analysis Protocol; Version 5.7, March 2008 Agilent) [7]. GENESPRING software version 7.3.1 (Agilent Technologies/Silicon Genetics) was used for the *in silico* analysis of microarray data [16]. Microarray signal intensities from laser microdissected female tissues were normalised against those of laser microdissected whole female controls, while signal intensities from testis samples were normalised against those of laser microdissected whole male controls. Macro-dissected male and female samples were normalised against whole male and female control samples, respectively.

Hybridized slides were scanned using an Agilent Microarray Scanner (B version) as Tiff files and processed with the Feature Extraction 9.5.3.1 Image Analysis program (Agilent) to produce standardised data for statistical analysis. All microarray slides were assessed for background evenness by viewing the Tiff image by Feature Extraction. Feature extracted data were analysed using GENESPRING (version 7.3.1; Agilent Technologies/Silicon Genetics, Redwood City, CA). Microarray data were normalised using a normalisation scenario for “Agilent FE one-color” which including “Data Transformation: Set measurements less than 5.0 to 5.0”, “Per Chip: Normalize to 50th percentile” and “Per Gene: Normalize to median”. Data sets were further analysed using published procedures based on one-colour experiments [17]. The gProcessedSignal values were determined in GENESPRING using Agilent's Feature Extraction software including aspects of signal/noise ratio, spot morphology and homogeneity. Thus, gProcessedSignal represents signal after localised background subtraction and includes corrections for surface trends. Features were deemed Absent when the processed signal intensity was less than two fold the value of the processed signal error value. Features were deemed Marginal when the measured intensity was at a saturated value or if there was a substantial amount of variation in the signal intensity within the pixels of a particular feature. Features that were not Absent or Marginal were deemed Present. Data points were included only if Present or Present/Marginal and probes or contigs retained if all data points were Present or Present/Marginal.

Microarray data have been submitted to the Gene Expression Omnibus public database, under accession numbers GPL10875 and GSE23942.

### Gene ontology analysis

Updating of annotation was performed on 20 October 2010 on all of the differentially expressed genes (6,295) detected in the four tissue types, using the Blast2GO online annotation pipeline [18]. BlastX was performed using the NCBI blast server URL (http://www.ncbi.nlm.nih.gov/Blast.cgi), ExpectedValue 1.0.E-3, and QBlast mode. The Gene Ontologies were assigned based on the hits received from the Blast using default settings. InterPro annotations were identified and retrieved domain/motif information used to transfer GO terms with already existent GO terms. Genes with GO annotation were submitted for Enzyme code and KEGG map assignment. The sequence description based is on BlastX analysis, sequence length and the number of hits (Table S3).

### Real-time PCR validation of microarray data

Gene expression patterns determined by microarray analysis were validated using real-time PCR. A subset of genes was selected for each tissue for validation. The selections were made based on the high level of expression and the importance of function in each tissue. Forward and reverse primers (Sigma-Aldrich, Castle Hill, Australia) were designed for 14 selected contigs (Table S4). All total RNA samples were DNase-treated (Promega, Annandale, Australia) before complementary DNA (cDNA) synthesis [11]. Real-time PCR was performed and analysed using previously described protocols [19]. DNA segregation ATPase [16] (TC15682- Smp176580, Contig809759.1) was used as housekeeping gene for the real time PCR reactions. Real-time PCR and microarray data were compared using GraphPad Prism Version 5.00 (GraphPad Software, San Diego, USA) [16], [20]. Data from microarray and real time PCR analyses were examined to ascertain if they fitted normal distributions, using the D'Agostino and Pearson omnibus and the Shapiro-Wilk normality tests.

## Results

### Laser microdissected tissues

**Figure 2:**
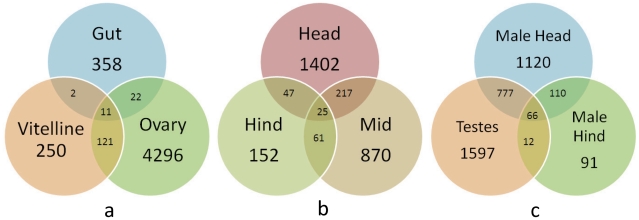
Genes enriched in specific tissues of adult *S. mansoni*. Venn diagram showing the numbers of *S. mansoni* genes up-regulated at least 2-fold in (a) microdissected female tissues, (b) macrodissected female tissues, (c) macro-dissected male tissue and testes.

Signal from female microdissected tissues (Figure 1), normalised against the whole female microdissected sample and subsequent filtering for flags revealed 15,109 contigs of which 5,060 were expressed 2-fold or higher than the female control in at least one of the three target tissues. The number of genes identified as being enriched in each tissue is shown (Figure 2a, Table 1, Table 2, Table 3, Table 4, Table S5). The majority of enriched genes were detected in the ovarian sample. A total of 121 genes were enriched for both vitellaria and ovary, which was as expected, considering the ontogenetic and functional similarities of the two tissues. Fewer genes were enriched in both the gastrodermis and either of the two female germinal tissues. Three hundred and fifty eight (358) genes were enriched for the gastrodermis only; of these 112 were significantly up-regulated (P<0.05). A representative list of genes highly expressed in the gastrodermis is shown (Table 1). Among those highly expressed were digestive proteases with diverse mechanistic activity, transmembrane transporters, lysosomal proteins and many unknown proteins. A large proportion (>40%) of the contigs enriched in the gut encoded hypothetical proteins with or without conserved domains, or unknown proteins with no sequence similarity to any known proteins from other organisms.

**Table 1:** A selection of differentially up-regulated genes of the female gastrodermis normalised to the whole female control.

*Systematic name	Gene name	Fold change	Contig	Protein description
Q2_P17138	Smp_127380	147.3	C918435.1	huntingtin associated protein-1 domain, putative
Q2_P37910	Smp_014610	8.83	C919425.1	conserved hypothetical protein
Q2_P27373	Smp_166170	7.98	C900002.1	hypothetical protein
Q2_P35922	Smp_128430	6.58	C915671.1	cd36 antigen, putative
Q2_P17781	Smp_095980	4.32	C920836.1	superoxide dismutase precursor (EC 1.15.1.1), putative
Q2_P04863	Smp_151910	4.1	C808892.1	multidrug resistance protein 1, 2, 3 (p glycoprotein 1, 2, 3)
Q2_P17708	Smp_138570	4.04	C920388.1	spore germination protein, putative
Q2_P35898	Smp_014570	3.91	C915640.1	saposin- saposin -containing protein
Q2_P37714	Smp_085010	3.41	C919093.1	putative cathepsin B,
Q2_P07155	Smp_136730	3.4	C812496.1	cathepsin D2-like protein
Q2_P35878	Smp_194850	3.23	C915617.1	Niemann Pick type C2 protein homolog
Q2_P40679	Smp_006760	3.09	JAP06514.C	fasting-inducible integral membrane protein tm6p1-related
Q2_P23552	Smp_152050	3.08	C807737.1	tetraspanin 18, isoform 1, putative
Q2_P30192	Smp_004190	2.76	C904653.1	cationic amino acid transporter, putative
Q2_P19424	Smp_138380	2.57	C802143.1	leucine aminopeptidase- related
Q2_P19417	Smp_139160	2.33	C802136.1	cysteine protease family C1-related
Q2_P03089	Smp_162770	2.7	C806088.1	lysosome-associated membrane glycoprotein
Q2_P03953	Smp_173160	2.3	C807425.1	zinc metalloprotease, putative
Q2_P04728	Smp_137170	2.25	C808691.1	plasma membrane calcium-transporting atpase, putative
Q2_P25963	Smp_159350	2.22	C811300.1	platelet binding protein-related

*The systematic naming of probes follows [14].

**Table 2:** A selection of differentially up-regulated genes of the vitelline tissue normalised to the whole female control.

*Systematic name	Gene name	Fold change	Contig	Protein description
Q2_P25336	Smp_164320	12.67	C810082.1	hypothetical protein
Q2_P41174	Smp_041540	4.02	JAP07007.C	hormone receptor 4 (dHR4), putative
Q2_P39884	Smp_056350	3.33	JAP08870.C	mitotic spindle assembly checkpoint protein mad2-
Q2_P40473	Smp_170990	3.05	JAP07382.S	vinculin, putative
Q2_P39851	Smp_174820	2.85	JAP08581.C	serine/threonine kinase, putative
Q2_P36026	Smp_173320	2.58	C915812.1	tubulin tyrosine ligase-related
Q2_P37617	Smp_169380	2.53	C918667.1	protein-tyrosine phosphatase, putative
Q2_P36808	Smp_162900	2.37	C917244.1	tyrosine-protein kinase pr2, putative
Q2_P38755	Smp_148680	2.28	JAP01788.S	DNA double-strand break repair rad50 ATPase,
Q2_P27568	Smp_076950.2	2.24	C900263.1	solute carrier family 33 (acetyl-CoA transporter)
Q2_P26890	Smp_008580	2.23	C812627.1	sugar transporter, putative
Q2_P31891	Smp_124110	2.13	C907823.1	smad nuclear interacting protein, putative
Q2_P35951	Smp_137080	2.11	C915708.1	multidrug resistance protein 1, 2, 3
Q2_P33700	Smp_158400	2.04	C911301.1	receptor protein tyrosine phosphatase r (pcptp1),

*The systematic naming of probes follows [14].

**Table 3:** A selection of differentially up-regulated genes of the ovary normalised to the whole female control.

*Systematic name	Gene name	Fold change	Contig	Protein description
Q2_P35767	Smp_141900	70.62	C915481.1	expressed protein
Q2_P17568	Smp_144080	69.56	C919966.1	proprotein convertase subtilisin/kexin-related
Q2_P35553	Smp_150350	57.65	C915220.1	synaptotagmin, putative
Q2_P22372	Smp_080360	52.15	C806137.1	hypothetical protein
Q2_P06908	Smp_011870	50.87	C812082.1	hypothetical protein
Q2_P32715	Smp_026400	38.6	C909489.1	thyrotroph embryonic factor related
Q2_P34493	Smp_027920	24.25	C912754.1	tubulin alpha chain, putative
Q2_P32366	Smp_082490	17.99	C908755.1	cyclin B, putative
Q2_P02801	Smp_153660	15.49	C805565.1	cyclin d, putative
Q2_P39175	Smp_067260	14.77	JAP01374.C	transforming growth factor-beta receptor type I, putative
Q2_P41215	Smp_145580	14.09	JAP08203.C	progesterone-induced-blocking factor, putative
Q2_P14033	Smp_160630	13.32	C911654.1	DNA repair protein rad51 homolog 3, r51h3, putative
Q2_P24711	Smp_004060	11.98	C809241.1	cell division cycle 20 (cdc20) (fizzy), putative
Q2_P29040	Smp_162710	11.46	C903052.1	aurora kinase-related
Q2_P40215	Smp_135900	9.43	JAP01762.S	DNA double-strand break repair rad50 ATPase, putative
Q2_P00792	Smp_160650.2	5.43	C801902.1	smad1, 5, 8, and, putative
Q2_P28420	Smp_145900	4.69	C902171.1	adam, putative
Q2_P14621	Smp_089700	4.16	C912832.1	integrin beta subunit, putative
Q2_P36767	Smp_144390	3.21	C917178.1	transforming growth factor-beta receptor type I and II, putative
Q2_P05400	Smp_165220	3.15	C809991.1	embryonic ectoderm development protein, putative
Q2_P11728	Smp_033950	2.66	C907151.1	Smad4, putative
Q2_P18126	Smp_157540	2.54	C800274.1	smad, putative
Q2_P22966	Smp_072370	2.31	C806933.1	major egg antigen (p40)

*The systematic name of probes follows [14].

**Table 4:** A selection of differentially up-regulated genes of testis normalised to the whole male control.

*Systematic name	Gene name	Fold change	Contig	Protein dscription
Q2_P28420	Smp_145900	32.89	C902171.1	ADAM, putative
Q2_P39027	Smp_165970	31.26	JAP00180.C	acidic fibroblast growth factor intracellular binding protein, putative
Q2_P38111	Smp_143970	26.69	C920217.1	kelch-like protein
Q2_P35735	Smp_157820	26.09	C915438.1	ataxia telangiectasia mutated (atm), putative
Q2_P30827	Smp_074830	20.54	C905692.1	tubulin tyrosine ligase-related
Q2_P40105	Smp_067260	19.78	JAP11452.C	transforming growth factor-beta receptor type I, putative
Q2_P02282	Smp_032490	19.72	C804679.1	tropomyosin, putative
Q2_P14033	Smp_160630	15.73	C911654.1	DNA repair protein rad51 homolog 3, r51h3, putative
Q2_P38558	Smp_169970	14.78	JAP06742.C	Paramyosin, putative
Q2_P39506	Smp_136210	10.12	JAP03797.C	testis development protein nyd-sp29, putative
Q2_P34493	Smp_027920	9.08	C912754.1	tubulin alpha chain, putative
Q2_P25694	Smp_167000	8.73	C810809.1	testis specific protein, putative
Q2_P32904	Smp_042740	8.47	C909800.1	sperm associated antigen 6
Q2_P06063	Smp_167610	7.02	C810843.1	multidrug resistance-associated protein 4 (mrp/cmoat-related abc transporter
Q2_P12548	Smp_139730	5.04	C908741.1	intraflagellar transport 81
Q2_P21892	Smp_075140	3.96	C805511.1	spermatogenesis associated 18
Q2_P10649	Smp_058320	3.68	C904995.1	nuclear autoantigenic sperm protein (nasp), putative
Q2_P20054	Smp_078040	3.45	C802934.1	tubulin beta chain, putative
Q2_P39599	Smp_152680	3.41	JAP05136.C	epidermal growth factor receptor, putative
Q2_P22159	Smp_104730	3.17	C805862.1	DNAj homolog subfamily B member 4, putative

*The systematic naming of probes follows [14].

For the testes, after filtering the microarray data for flagged signal and normalising against the whole male control, a total of 16,334 contigs were revealed, of which 2,171 were up-regulated 2 fold or higher than the whole male control (Table S5). We also compared gene transcription between the male and female reproductive tissues. The ovary had more genes in common with testes than with the vitelline tissue, while all reproductive tissues expressed 35 enriched contigs in common (Table S6, Figure S1).

GO distribution for each tissue type is shown in Figure S2 using level 2 annotations. A pie chart representation and the number of contigs at level 2 annotation are shown for either “Biological Process” or “Molecular Function”. The top 10 GO annotations, based on overall contig numbers for each ontology, are shown (Figure S3). Results are shown for either “Biological Process” or “Molecular Function”. Annotations for the 6,295 contigs that were differentially expressed in any 1 of the 4 tissue types isolated by LMM, as determined by microarray analysis, are shown (Table S5).

### Macro-dissected female tissues

Of the 41,799 probes on the microarray, 18,359 were present in all the macro- dissected female tissue samples when normalised against the whole female control and filtered for significant signal. Three thousand one hundred and nine genes were up-regulated two-fold or more in at least one region of the parasite, compared with the control sample, and their presentation in each region is shown (Figure 2b). The head and middle regions had fewer genes in common with the hind region (Table S7, Table S8, Table S9) and this may be due to the dominance of vitelline tissue in the hind region of adult female schistosomes. The microdissected ovarian sample and the female middle region showed 574 genes enriched in common, which is probably due to the anatomical location of the ovary within this region (Table S1), but the vitelline tissue had only 4 common genes with the hind region. This latter result is interpreted as being due to the dominance of vitelline gland transcriptional activity in females overall.

There were 3,773 genes up-regulated in manually dissected male tissues and the testes when compared with the whole male worm. Genes that were up-regulated two fold or higher in each male tissue sample are shown (Figure 2c and Table S10, Table S11). The testes were included in the male hind region during manual dissection, but the analysis showed that the testis had more genes in common with the head than the hind region. This may reflect similar gene expression in tissues bordering or surrounding the testes, or may reflect the gross manual dissection procedure. Either way, the accuracy of LMM provides the best isolation method for this type of tissue, and this type of experimental approach.

### Real-time PCR validation of the microarray data

The D'Agostino & Pearson omnibus and the Shapiro-Wilk normality tests showed that the data were not normally distributed. Therefore, Spearman correlation was used [20] to compare the real-time and microarray data. Comparison of 31 data pairs using the Spearman correlation showed a statistically significant correlation (P<0.0001, alpha = 0.05) between the real-time and microarray data (Figure S4).

### Comparison of expression profiles of *S. japonicum* and *S. mansoni* adult tissues

In our previous study, we investigated expression profiles of three female tissues of *S. japonicum* using a different microarray platform [11]. To compare more directly the expression profiles of these tissues in *S. japonicum* and those of *S. mansoni* presented here, BLASTn searches of the two datasets were performed to identify potential homologues enriched in tissue for both species (Table S2).

Approximately 72% of the contigs on the microarray used in the previous investigation of *S. japonicum* tissue-specific expression corresponded to putative orthologues of genes represented on the current microarray, with homology greater than 75% and covering more than 40% of the sequence length (e-value<1E-10). When the previous reported *S. japonicum*/*S. mansoni* microarray [11] was compared to that used here on the basis of design, approximately 95% of contigs derived from *S. mansoni* (TC) had homologues on the current microarray, whereas only approximately 33% of contigs derived from *S. japonicum* ESTs on that platform had strong homology to sequences on the current microarray. Overall there are strong similarities between the combined (*S. japonicum* and *S. mansoni*) microarray [11] and the microarray used in the current study [14] which was based on a more recent EST assembly.

The considerable overlap between the two microarrays allowed us to make direct comparisons of expression profiles of tissues of the two schistosome species. Of the 393 up-regulated genes in *S. mansoni* gastrodermal tissue, 180 had homologues in the microarray used in the previous *S. japonicum* study [11], and 31 of these were transcriptionally enriched in the *S. japonicum* gastrodermis. Similarly, of the 384 enriched genes in *S. mansoni* vitelline samples, 188 had homologues in the combined microarray, of which 33 were enriched also in the *S. japonicum* vitelline tissue. Four thousand four hundred and fifty (4,450) upregulated genes in *S. mansoni* ovary had 2675 homologues, and 917 of these were enriched in the *S. japonicum* ovary (Table S5).

## Discussion

In this paper we present transcriptomic data for four tissues in adult worms of *Schistosoma mansoni*. This study complements our previous description of tissue-specific transcriptomics for the related species *S. japonicum* [11], but, additionally, provides the first expression analysis of the testes of a schistosome. As a means to defining the transcriptome of other less accessible tissues, we have also incorporated data from crude dissection of adults. Although this latter dataset cannot replace detailed localisation investigations of specific tissues, it can be used to give a crude overview of sites of expression of genes.

For this study, we pooled multiple schistosome parasites, from separate infections, so as to allow the microdissection of sufficient volumes of tissues necessary to obtain sufficient total RNA for downstream analysis. This approach limits any impact that variation between individual parasites may have on gene expression. Due to extreme technical constraints in the LMM method, the numbers of strict biological replicates was restricted, but we are confident that the use of pooled individual parasites limits any drawbacks normally overcome with biological replicates. Our data fulfils the criterion of biological relevance, that is, genes, for which we know function and sites of expression, were largely expressed in the tissue in which we expected them to be. Thus, while there is a limitation brought about by the sheer immensity of the undertaking, we argue that the results are robust. Further surveys will augment our dataset.

**Figure 3:**
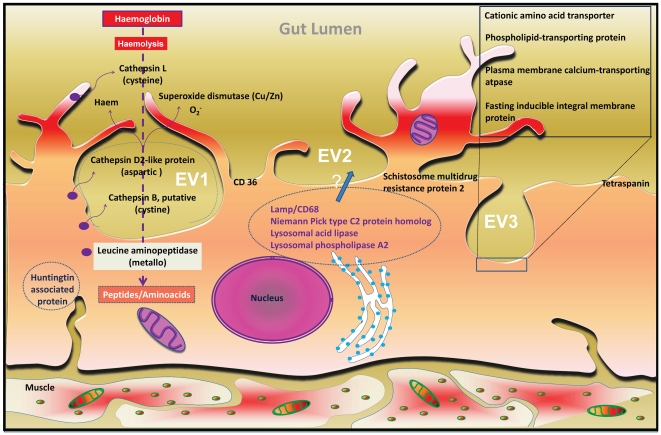
Diagrammatic representation of the gastrodermis of female *S. mansoni*. The diagram is based on an illustration originally published by Morris [52]. The illustration shows some highly enriched genes identified in this transcriptomic survey (Table 1, Table S3). Many of the genes products are putatively released into, or onto the membrane of epicellular vacuoles (EV) present at the luminal surface of the syncytial epithelium. Three EVs are depicted (EV1–3), each showing different physiological activities proposed to occur in the vacuoles. EV1 depicts haemoglobinolysis pathways. Many proteases are released into the EV, resulting in catabolism of haemoglobin into amino acids or small peptides for uptake. Haem, a byproduct of catabolism, is sequestered into haematin (see illustration in [53]) for subsequent egestion. EV2 depicts a potential series of molecules associated with lysosomal functions, which has been transferred to these vacuolar compartments. EV3 shows a series of membrane proteins, involved in a variety of functions, notably transmembrane transport of metabolites, which would be preferentially associated with the epicellular vacuoles. All three physiological activities may occur in the same vacuole, probably concurrently.

Gene ontology analysis performed on the differentially expressed genes of all four tissue types gave insight into which common biological processes and molecular functions are present in the individual tissues. For all tissues examined here, most differentially expressed genes were involved in catalytic activity and binding functions. For the gastrodermis, probes for genes encoding cystine-type endopeptidase activity followed by RNA binding were well represented. With regard to biological processes and molecular function (Figures S2, S3), genes related to proteolytic process were, as expected, the most abundant in the gastrodermis (Figure 3), followed by genes for RNA dependent DNA replication and genes related to auxin biosynthesis process, such as those encoding protein kinases, RNA and DNA helicases, and ATP-dependent transporters. The expression profile of the gastrodermis thus reflects the digestive and absorptive nature of the tissue as well as its high synthetic activity.

The gastrodermis transcribes genes encoding a range of endo- and exo-peptidases [21]–[24], saposins, structural genes, and numerous genes associated with intracellular compartments (Table 1, Table S5, Figure 3). Full knowledge of the proteolytic repertoire of the schistosome gut, however, is limited because we are largely ignorant about the expression repertoire of the proximal region of the gut, the oesophageal gland and oesophagus. This lack of data arises from the minute size of this gland in female schistosomes [12]. The oesophageal gland, a syncytial mass surrounding the tegument-like foregut, is thought to be responsible, among other activities, for lysis of red cells after their ingestion by the parasite [12], [25]. Our crude analysis of the expression profiles of the anterior regions of females suggest the expression of candidate genes that might contribute to host cell lysis, including a number of saposins and a diversity of apical peptidases (Table S7). Full characterisation of the haemoglobinolytic cascade in the entire schistosome gut requires detailed transcriptional analysis of the oesophageal gland. To address this, we have begun to analyse the expression activity of the male oesophageal gland to partially address this information deficit.

Our two transcriptomic surveys have shown strong overlap in the genes enriched for the gastrodermis of *S. japonicum* and *S. mansoni* [11] (Table S5). We detected a range of molecules encoding proteins involved in lipid binding, uptake or metabolism, such as saposins (for *S. mansoni*, these include Smp_130100 Smp_105420.1 Smp_014570 Smp_028870). As schistosomes are unable to synthesise sterols and lipids [1], the abundance of molecules for fatty acid transport emphasises their nutritional dependency on the host. Niemann Pick type C2 protein homologue (NPC2) genes (Smp_194850) (Figure 3, Table S5) are enriched for the gastrodermis for both schistosome species, as well as for the ovary of *S. mansoni* (Table S5), an observation consistent with the detection of abundant NPC2 in the secretome of *S. mansoni* eggs [26]. NPC2 is a lysosomal cholesterol-binding protein which, in conjunction with NPC1, helps in the trafficking of cholesterol and other sterols and glycolipids in eukaryotic cells [27], [28]. While NPC2 was found to be enriched in the gastrodermis of *S. mansoni*, its putative binding partner, NPC1, was enriched for the head region of females (Table S7), a region that contains the secretory oesophageal gland.

The gastrodermis of schistosomes bears numerous epicellular vacuolar compartments [22] that are suggested to function in a lysosomal capacity. We detected numerous genes that in mammals are associated with membrane trafficking through endocytosis, endocytic compartments and lysosomes in the gastrodermis of *S. mansoni*. Among these enriched genes were a homologue of human Huntingtin associated protein 1 (HAP 1) (Smp_127380) [29], and numerous lysosomal molecules including 1-o-acylceramide synthase (Smp_166530.1) (lysosomal phospholipase A2 or lcat-like lysophospholipase) and lysosomal acid lipase-related protein (Smp_146180). In addition, a homolog of a lysosome-associated membrane glycoprotein (LAMP)/CD68 (Table 1, Figure 3) (Smp_162770) was enriched for the gastrodermis of both schistosome species [11] and is also abundant in the ovary of *S. mansoni*. LAMP family members sit in the membranes of lysosomes, where they cover the majority of the lysosomal luminal surface [30], [31]. Other LAMPs occur in the plasma membrane of cells such that much of the LAMP protein is extracellular [32], [33]. If the epicellular vacuoles of the schistosome gastrodermis function as part of the endocytic or lysosomal system, then such molecules as schistosome NPC2 and LAMP may function in these luminal components of the gastrodermis, and may represent accessible drug or vaccine targets.

Gene ontology analysis of reproductive tissues, including the vitellaria, of *S. mansoni* showed that the greatest number of enriched genes had molecular functions associated with ATP and protein binding, while biological processes associated with auxin metabolism, signal transduction and transmembrane transport were also prominent. Expression profiling of the three reproductive tissues indicated that in all some 35 genes were enriched for all three tissues. Many of these genes encode products with no annotation, and thus no assigned function. Of genes that did have annotations, some encoded proteins involved in cell cycle and DNA repair, as would be expected for high turnover tissues. The ovary had more genes in common with the testes, than with the vitellaria (Table S6).

Egg shell precursor proteins were highly up-regulated in the vitellaria relative to the ovary, with one (Smp_000430.1) being enriched some 78-fold in the former tissue. Hormone receptor 4 (dHR4) (Smp_041540), which was up-regulated 4-fold in *S. mansoni* vitelline tissue (Table 2), is a nuclear receptor which is a transcription regulator [34]. King-Jones and colleagues [35] have suggested that dHR4 could be one of the factors that coordinate hormone-mediated growth and maturation in *Drosophila*.

In the ovary, we found a number of genes encoding proteins that in mammals are thought to dock sperm during fertilisation, such as the alpha and beta integrins [36], [37] and an ADAM (A Disintegrin And Metalloproteinase) (Smp_145900) [36] (discussed below). Tyrotrophic embryonic factor related protein (Smp_026400) was one of the highest transcripts up-regulated (30-fold) in the ovary and is known to regulate transcription [38]. Several other proteins involved in DNA repair and cell cycle were enriched in the ovary. Cyclins and their protein kinases play a key regulatory function in the cell cycle. Cyclins, mainly Cyclin B (Smp_082490, Smp_047620), which is associated with the mitotic phase [39], were highly enriched in both ovary (17.99 fold) and testis (19.3 fold) (Table 3, Table S5).

An ADAM homologue was the second most highly expressed gene in the testes of *S. mansoni* (Table 4, Table S5). Its expressed product plays a role in fertility by helping sperm-egg adhesion. Other than fertilisation, ADAMs are also involved in neurogenesis, myogenesis and inflammatory processes [36], [40]. Among the 2171 genes enriched 2-fold or greater in the testes were molecules specifically related to testicular function, including the testis development protein nyd-sp29, testis specific protein, sperm-associated antigen 6, spermatogenesis-associated protein, and nuclear autoantigenic sperm protein (nasp) (Table 4 and Table S5).

Schistosomes are known to use well-defined signalling pathways for development, and for between-gender and host-parasite cross-talk [41], [42]. Females need the presence of mature male worms for their development, and oogenesis and vitellogenesis [41], [42]. Our data revealed enrichment of transmembrane receptors involved in protein phosphorylation, major components of signaling pathways, including serine threonine kinase and tyrosine kinase, in gonadal tissues of males and females (Table S3, Table S5, Table S6). Furthermore, all key molecules of the TGFβ-related pathway, a pathway important for female reproductive activity [42], [43], were enriched in the ovary, including transforming growth factor-beta receptors (TGFβR) and putative Smad proteins including Smad4 (Smp_033950) and Smad 1, 5, and 8 (Smp_160650.2) (Table 2). Smads were also enriched in vitellaria, whereas TGFβR1 was up-regulated in the testes.

We performed relatively crude dissections to assess gene expression in different regions of adult *S. mansoni*. The anatomy of schistosomes is highly ordered with specific tissues located in different regions of the parasites (Table S1). We reasoned that this crude dataset could serve as a means for further fine-tuning sites of expression of genes that are expressed in tissues difficult to isolate with current technologies. These data have been presented here with the caveat that localisation will need to be further confirmed with other localisation methods including *in situ* hybridization and further LMM.

Of particular interest was the expression profile of the female head region. This region contains the oesophageal gland, the tissue likely to express enzymes initiating haemoglobinolysis and red cell lysis. Many proteases that were not common to the gastrodermal tissue were up-regulated in the head region. Genes encoding proteins associated with neuronal tissue were also up-regulated in this region (Table S7), reflecting the location of the circum-oesophageal ganglia. Abundant molecules with neuronal function in the head region included neurotransmitter gated ion channel proteins (Smp_096480 Smp_176310 Smp_037960) and a vesicular acetylcholine transporter (Smp_046120). Cerebellin (Smp_105050), a neuropeptide known to be found mainly in central nervous system related to neuromodulatory function [44], and stomatin (Smp_162440), involved in signal transduction from mechanoreceptors [45], were among some of the other enriched genes. Kuzbanian (Smp_160620.1), a molecule related to neural development in mammals [1], was up-regulated in the head region of adult females.

Chalmers and colleagues [46] have described 28 *S. mansoni* venom allergen-like proteins (SmVALs) which contain a sperm coating protein domain. No specific function has been ascribed to these proteins, but they may have diverse biological roles including counteracting the host immune response, host invasion and host parasite interactions. We found SmVAL- 6 (Smp_124050.1) to be 31-fold enriched in the female head region compared with the female control, although this molecule was previously reported to be predominately associated with adult males [46]. SmVAL6 was recently identified as a microexon gene [1], [47] and is one of at least 45 *S. mansoni* genes thought to undergo alternative splicing, resulting possibly in the generation of multiple gene products [47]. Another distinct VAL, SmVAL-13 (Smp_124060) was enriched 14-fold head region of males (Table S7 and Table S10).

In summary, this is the second study of tissue specific transcriptomics of the digestive and reproductive systems in schistosomes and the first such study of *S. mansoni* which, additionally, includes an expanded tissue analysis of the male reproductive tissues. In addition to established genes associated with digestive function, we have provided localisation data for new putative transporters, receptors, cytoskeletal elements and motor proteins. Furthermore, many other genes without any formal annotation were prominent transcripts in the gastrodermis. Similarly, many known genes involved in DNA fidelity, oocyte development and egg shell formation were identified in the reproductive tissues along with numerous unidentified molecules. Some of these as yet unknown genes may be involved in transport processes, signal transduction and receptor function, and include cytoskeletal elements and motor proteins. The regulation of the cell cycle and gene expression is another important function that must be maintained in the reproductive tissues of schistosomes. The findings of this study provide a valuable resource for the parasitology community and add a new dimension to the already available stage-specific transcriptomic data for *S. mansoni*. The presented data represent a resource that could be used effectively in reverse vaccinology allowing the targeting of hidden antigens in the gastrodermis that are exposed to the blood meal, a strategy used effectively for other blood feeding parasites [48], [49], [50], [51], and in the search for new anti-schistosome drug target candidates targeting the control of fertility and fecundity.

## Supporting Information

Figure S1 Venn diagram showing genes up-regulated in microdissected male and female reproductive tissues of *S. mansoni*.
(1.01 MB TIF)

Figure S2 Gene Ontology distribution for male and female microdissected tissues. GO distribution for Biological Process level 2 (left) or Molecular Function level 2 (right) for *S. mansoni* male and female microdissected tissues. The number of genes in each category is in brackets.
(2.16 MB TIF)

Figure S3 Top 10 Gene Ontology annotations by gene number for Biological Process and Molecular function in *S. mansoni* male and female microdissected tissues. The categories are listed, with the number of sequences in each category.
(2.69 MB TIF)

Figure S4 Real-time PCR validation of microarray. Validation is shown using a subset of differentially expressed genes in *S. mansoni* female gastrodermis, ovary, vitelline tissues compared to the female control and testes compared to the male control tissue. The real-time PCR data, expressed as fold changes (normalised to control tissues, either male or female all tissues), are presented as bar graphs, while the corresponding microarray data (fold changes) are shown below in numbers.
(0.41 MB TIF)

Table S1 Major tissue components of female macro-dissected regions.
(0.03 MB DOC)

Table S2 Comparison of the microarray used in the *S. japonicum* transcriptomic study [11] and the microarray used in the current study using BioEdit BLASTn. Column A: Name of EST from either *S. japonicum* (ContigXXX) or *S. mansoni* (TCXXX) used from the study of Gobert et al (2009) [11]. Column B: Length of the EST from Column A. Column C: % of the length from EST of Column A that was homologous to the BLASTn from ESTs on the microarray from the current study. Column D: EST result from BLASTn results % nucleotide identity to the current study's microarray platform. Column E: % nucleotide identity, the extent to which two sequences were invariant. Column F: Nucleotide alignment length. Column G: The number of nucleotides that did not match. Column H: A space introduced into an alignment to compensate for insertions and deletions in one sequence relative to another. Columns I–J: The input sequence with which all of the entries in a database were to be compared. Column I: Coordinate of the query start. Column J: Coordinate of the query end. Columns K–L: The database which the input sequence was compared with. Column K: Coordinate of the subject start. Column L: Coordinate of the subject end. Column M: Expectation value. The number of different alignments with scores equivalent to or better than S that are expected to occur in a database search by chance. Column N: Bit Score, The value S′ is derived from the raw alignment score S in which the statistical properties of the scoring system used have been taken into account.
(3.41 MB XLS)

Table S3 BlastX results of the complete list of differentially upregulated genes in *S. mansoni* female and male microdissected tissues (2 fold or higher than the microdissected whole female control for ovary, vitelline and gastrodermis and 2 fold or higher than the microdissected male control for testes). Column A: EST name from current study microarray platform [14]. Column B: BLASTx description. Column C: Length of the EST. Column D: Number of homologous matchs from BLASTn. Column E: eValue- Expectation value. The number of different alignments with scores equivalent to or better than S that are expected to occur in a database search by chance. Column F: mean % sequence identity, the extent to which two sequences are invariant. Columns G–M: Top homologous hit details. Column G: Accession numbers for the homologous protein description. Column H: Homologous hit accession number. Column I: eValue- Expectation value. Column J: % sequence identity, the extent to which two sequences are invariant. Column K: Bit Score, The value S′ is derived from the raw alignment score S in which the statistical properties of the scoring system used have been taken into account. Column L: Alignment length between the query and the top homologous hit. Column M: Number of positive sequences. Column N: Number of Gene Ontology categories associated with the top homologous hit. Column O: Associated gene ontology codes. Column P: Associated gene ontology names. Column Q: Enzyme codes. Column R: Enzyme pathways. Column S: InterProScan codes. Column T: Additional InterProScan output information.
(5.71 MB XLSX)

Table S4 List of oligonucleotides used in real time PCR to validate the microarray data.
(0.03 MB DOC)

Table S5 Complete list of differentially upregulated genes in *S. mansoni* female and male microdissected tissues. Worksheet 1–3: Up-regulated genes in the female parasite sorted for each tissue type based on descending signal intensity, Gastrodermis, Ovary and Vitelline tissues, relative to the entire LMM-obtained control female tissues. Worksheet 4: Up-regulated genes in the male parasite Testes tissues, relative to the entire LMM male tissues. Column A: Systemic Name, unique identifier. Column B: Normalised signal intensity relative to control (all tissues) tissue. Column C: Normalised signal intensity of gastrodermis tissue. (Testes in worksheet 4). Column D: Normalised signal intensity of ovary tissue. (Blank in worksheet 4). Column E: Normalised signal intensity of vitelline tissue. (Blank in worksheet 4). Column F: Synonyms for unique identifier. Column G: Contig name. Column H: Homologue in Gobert et al, 2009 [11] study (genes found upregulated in same tissue between both [current] studies are highlight in blue). Columns I–M: Class, Annotation from Blast against GenBank on Jan 2008, Probe Sequence, SOURCE_ID and Description; all from original publication [14]. Columns N–AF: New Descriptions from BlastX performed on 20/10/2010. Column N: BLASTx description. Column O: Length of the EST. Column P: Number of homologous matchs from BLASTn. Column Q: eValue- Expectation value. The number of different alignments with scores equivalent to or better than S that are expected to occur in a database search by chance. Column R: mean % sequence identity, the extent to which two sequences are invariant. Columns S–Y: Top homologous hit details. Column S: Accession numbers for the homologous protein description. Column T: Homologous hit accession number. Column U: eValue- Expectation value. Column V: % sequence identity, the extent to which two sequences are invariant. Column W: Bit Score, The value S′ is derived from the raw alignment score S in which the statistical properties of the scoring system used have been taken into account. Column X: Alignment length between the query and the top homologous hit. Column Y: Number of positive sequences. Column Z: Number of Gene Ontology categories associated with the top homologous hit. Column AA: Associated gene ontology codes. Column AB: Associated gene ontology names. Column AC: Enzyme codes. Column AD: Enzyme pathways. Column AE: InterProScan codes. Column AF: Additional InterProScan output information.
(6.83 MB XLS)

Table S6 List of differentially upregulated genes common to *S. mansoni* male and female reproductive tissues (ovary, vitelline tissue and testes).
(0.11 MB XLS)

Table S7 Complete list of 1402 differentially upregulated genes (2 fold or higher than the whole female control) in the *S. mansoni* female head region.
(2.50 MB XLS)

Table S8 Complete list of 870 differentially upregulated genes (2 fold or higher than the whole female control) in the *S. mansoni* female middle region.
(1.75 MB XLS)

Table S9 Complete list of 152 differentially upregulated genes (2 fold or higher than the whole female control) in the *S. mansoni* female hind region.
(0.31 MB XLS)

Table S10 Complete list of 1120 differentially upregulated genes (2 fold or higher than the whole male control) in the *S. mansoni* male head region.
(2.66 MB XLS)

Table S11 Complete list of 91 differentially upregulated genes (2 fold or higher than the male control) in the *S. mansoni* male hind region.
(0.19 MB XLS)
