# Supplementary figures and images for: Gene Atlasing of Digestive and Reproductive Tissues in Schistosoma mansoni
Source: PLoS Negl Trop Dis. 2011 Apr 26;5(4):e1043. doi: 10.1371/journal.pntd.0001043 (PMC3082511; doi:10.1371/journal.pntd.0001043)

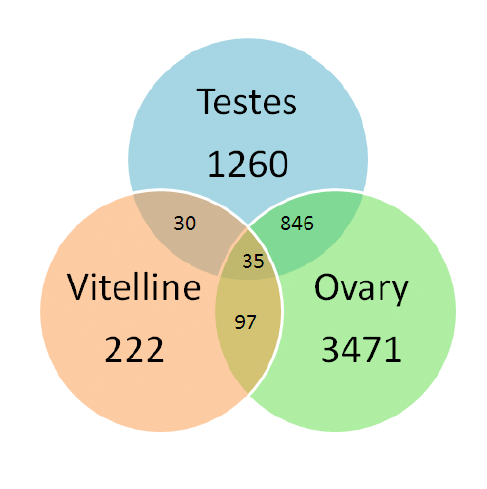

Supplement: Figure S1 — Venn diagram showing genes up-regulated in microdissected male and female reproductive tissues of S. mansoni. (1.01 MB TIF) [file pntd.0001043.s001.tif]

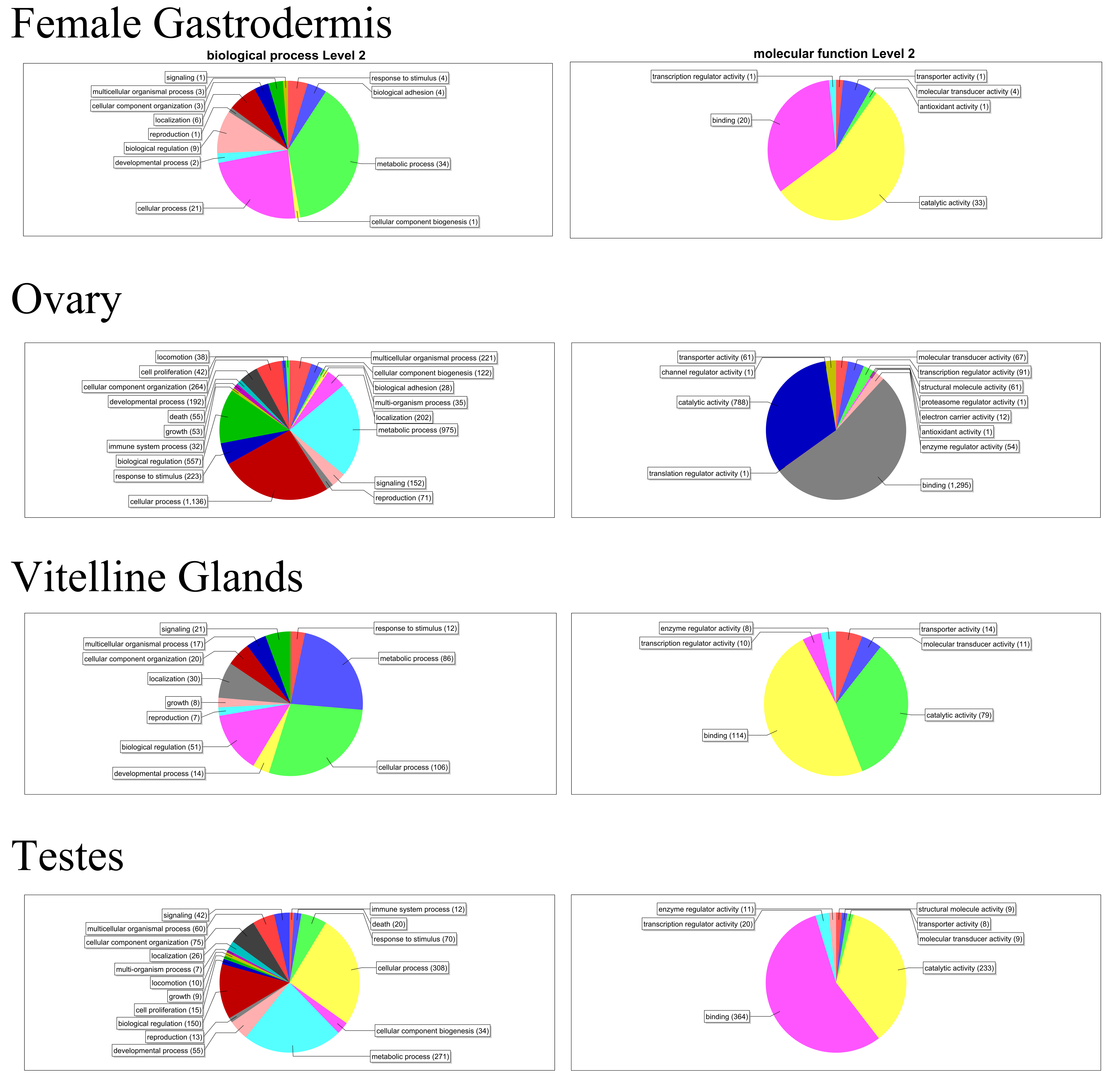

Supplement: Figure S2 — Gene Ontology distribution for male and female microdissected tissues. GO distribution for Biological Process level 2 (left) or Molecular Function level 2 (right) for S. mansoni male and female microdissected tissues. The number of genes in each category is in brackets. (2.16 MB TIF) [file pntd.0001043.s002.tif]

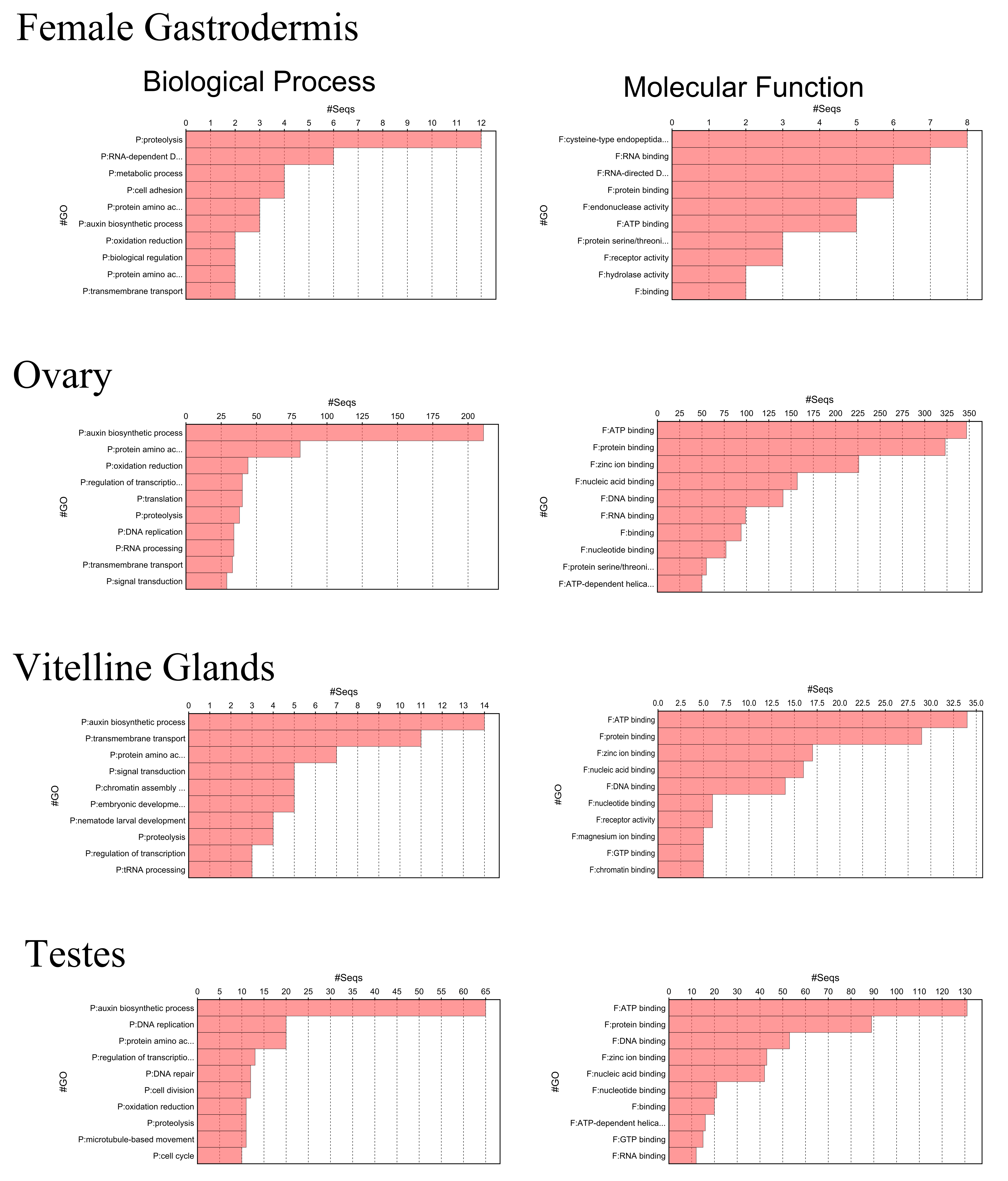

Supplement: Figure S3 — Top 10 Gene Ontology annotations by gene number for Biological Process and Molecular function in S. mansoni male and female microdissected tissues. The categories are listed, with the number of sequences in each category. (2.69 MB TIF) [file pntd.0001043.s003.tif]

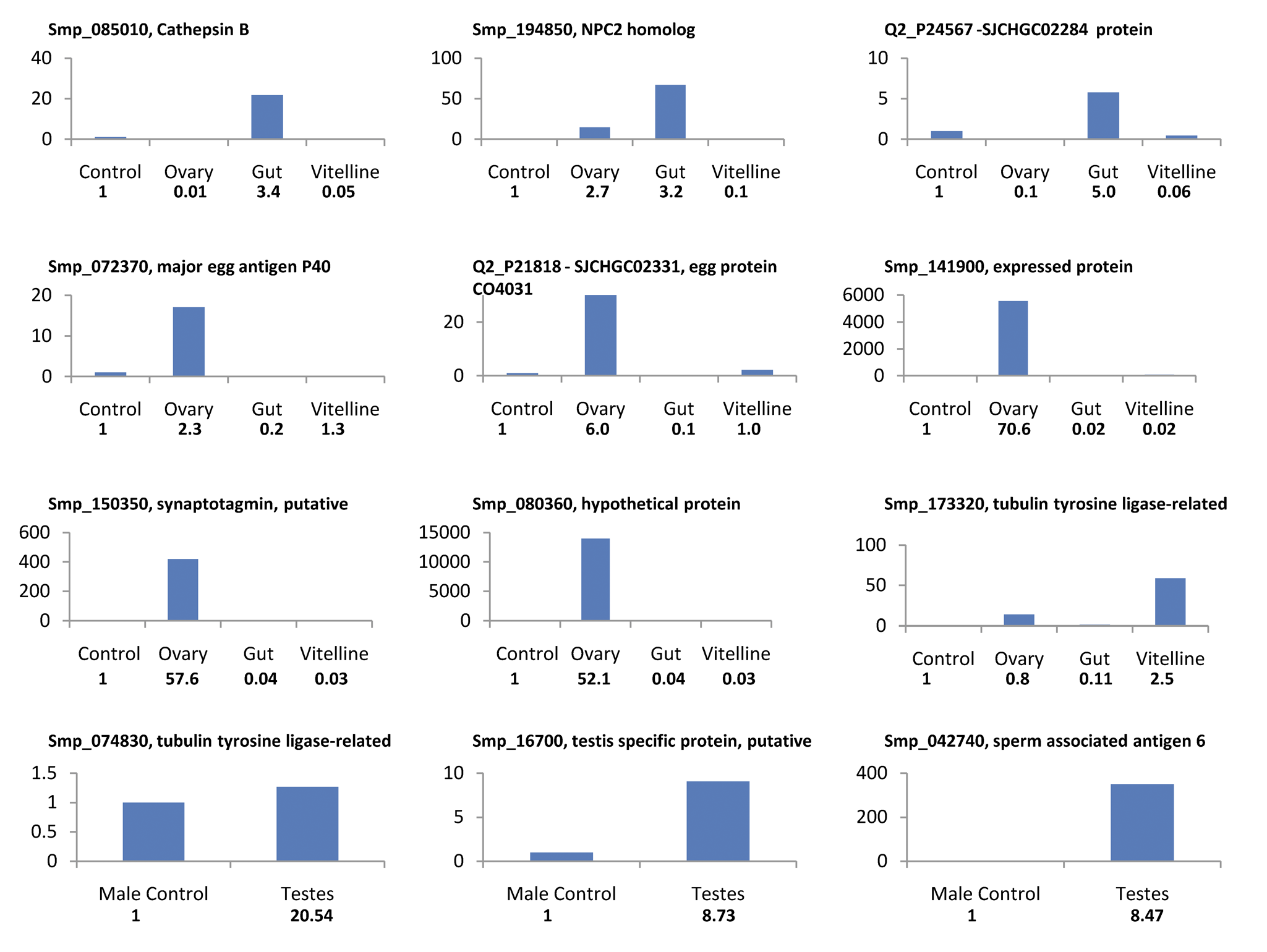

Supplement: Figure S4 — Real-time PCR validation of microarray. Validation is shown using a subset of differentially expressed genes in S. mansoni female gastrodermis, ovary, vitelline tissues compared to the female control and testes compared to the male control tissue. The real-time PCR data, expressed as fold changes (normalised to control tissues, either male or female all tissues), are presented as bar graphs, while the corresponding microarray data (fold changes) are shown below in numbers. (0.41 MB TIF) [file pntd.0001043.s004.tif]
