# Supplementary material for: Gene Atlasing of Digestive and Reproductive Tissues in Schistosoma mansoni
Source: PLoS Negl Trop Dis. 2011 Apr 26;5(4):e1043. doi: 10.1371/journal.pntd.0001043 (PMC3082511; doi:10.1371/journal.pntd.0001043)
Supplement: Table S1 — Major tissue components of female macro-dissected regions. (0.03 MB DOC) [file pntd.0001043.s005.doc]

**Table S1** Major tissue components of female macro-dissected regions

| **Head region** | **Middle region** | **Hind region** |
| --- | --- | --- |
| Fore gut  Oesophageal gland  Cerebral ganglion  Sensory receptors  Excretory system  Musculo-parenchymal system  Tegument | Ovary  Mehlis gland  Vitelline duct  Oviduct  Seminal receptacle  Uterus  Gastrodermis  Excretory system  Neuronal tissue  Musculo-parenchymal system  Tegument | Gastrodermis  Vitelline glands  Musculo-parenchymal system  Excretory system  Neuronal tissue  Tegument |
