# Supplementary material for: Gene Atlasing of Digestive and Reproductive Tissues in Schistosoma mansoni
Source: PLoS Negl Trop Dis. 2011 Apr 26;5(4):e1043. doi: 10.1371/journal.pntd.0001043 (PMC3082511; doi:10.1371/journal.pntd.0001043)
Supplement: Table S4 — List of oligonucleotides used in real time PCR to validate the microarray data. (0.03 MB DOC) [file pntd.0001043.s008.doc]

**Table S4** List of oligonucleotides used in real time PCR to validate the microarray data

| Gene name | Probe name | Primer pair sequence (5′-3′) | |
| --- | --- | --- | --- |
|  |  | Forward | Reverse |
| Smp_085010  Smp_194850  SJCHGC02284  Smp_072370  SJCHGC02331  Smp_141900  Smp_150350  Smp_080360  Smp_173320  Smp_145900  Smp_074830  Smp_136210  Smp_167000  Smp_042740 | Q2_P37714  Q2_P35878  Q2_P24567  Q2_P22966  Q2_P21818  Q2_P35767  Q2_P35553  Q2_P22372  Q2_P36026  Q2_P28420  Q2_P30827  Q2_P39506  Q2_P25694  Q2_P32904 | GGAATGGCATGGGATTATTG  CCCAAGAGACTGTAAAGGATGG  ACTCGGGATGTTCATCAAGG  TGAAGTTGAACAAGCCCAGA  TTTGTTGTGCTGCCAATTT  CATTTGCTATGCGTTCAAGC  TGGATTCACCAAATGAATGG  TCCACAACCTGGATTCATCA  GGTGTTTGGAGGTTGTCAGC  TGAAGAGCTAAACGCCTCGT  TCGGAGACCTACATTCTTGC  TGGGATTCACCTAAACGTCA  GACGATCATCCGCTAGCAA  CAGCTGATCTTGCTCAATCG | TCATGACATTCTGGTGTTGGA  ACAGGGAATTCAACGCAAAC  GCTGCTGGCCAACAAATAAT  GGATCAGCGCATAGAACAGC  TGGCATTAACCGGTAATAACATC  CGATCAAGGGCATTCTTTGT  CAGTGTAGGCCGGAGTCAAT  GCAATTGTGAGCTGCTTTGA  AATGGGCCAGAACACAGAGT  TTTCCCTAGCAGCAGCATCT  GATTATCCCATACTACAGGAGTTCTTT  AAATTCCAACGCATCGAAAC  CGTTGCGGGATAAGTATCAA  CGTCCTTAAGGCACACCAAT |
